# Precision Pain Management in Interventional Radiology: A Systematic Review -

**DOI:** 10.31661/gmj.vi.3922

**Published:** 2025-07-29

**Authors:** Mohammad Al Sayegh

**Affiliations:** ^1^ Department of Radiology in Mubarak alkabeer hospital, Graduate of Rcsi-dublin, FFR-RCSI University, Kuwait

**Keywords:** Precision Pain Management, Interventional Radiology, Chronic Pain, Personalized Treatment, Minimally Invasive Techniques, Clinical Outcomes, Evidence-based Protocols

## Abstract

Chronic pain, as a complex and individual phenomenon, still represents a significant issue in both the acute and chronic contexts. This study aimed to investigate the current state of pain management in IR procedures and identify potential areas for improvement in the standardization of pain management protocols. IR approaches are less invasive and focus on individualized treatment targeting the cause of pain, especially in chronic pain states. Precision pain medicine aims to tailor treatment plans based on algorithms grounded in evidence to address the needs of patients. This systematic review aims to assess the effectiveness of IR in precision pain management by analyzing its techniques, safety, and clinical outcomes. In light of the existing literature, this review aims to fill the knowledge gap, call for protocol harmonization, and demonstrate how IR can contribute to patient-centered care. The results are expected to contribute to the knowledge base, inform future research and clinical practice, and improve patients’ quality of life with multiple pain conditions.

## Introduction

Chronic pain is one of the most common experiences and has a significant impact on an individual’s quality of life, mobility, and mental health. Worldwide, it is estimated that pain management is a prevailing problem where approximately 20% of adults suffer from chronic pain, of which 10% experience severe pain that limits their ability to function [1]. For instance, in the United States of America, chronic pain is one of the leading causes of disability, costing up to $635 billion in healthcare costs and productivity loss every year. Recent studies in pain management show that many patients remain dissatisfied with the relief obtained, further stressing the importance of improved and individualized methods. Precision pain management is one of the novel approaches to medical practice, where different interventions are based on patient characteristics and pain mechanisms, comorbidities, and anatomy [2]. This approach enhances the effectiveness and, at the same time, security of treatments, turning from one-size-fits-all methods to personalized medicine approaches. Another area that can benefit from the precision pain treatment approach is interventional radiology (IR), which uses noninvasive procedures. Regional anesthetic techniques, like joint infiltration, lumbar blocks, radiofrequency nerve ablation (RFA), stimulation of the spinal cord (SCS), and neurolytic treatments are aimed at addressing the basic mechanisms of pain, especially in cases of chronic and chronic resistant pain [3]. Not only do these techniques enhance pain relief, but they also reduce the side effects of more radical surgical procedures.

However, the field of precision pain medicine is still in its infancy, with little consensus regarding standard and reliable methods to provide adequate pain management. Optimizing pain management in interventional practice depends on creating reliable algorithms based on clinical findings, imaging, and patient characteristics. Hence, this review aims to fill the existing literature gap in understanding IR’s scope, efficacy, safety, and outcomes in precision PM. This systematic review submits that interventional radiology can help enhance precision pain management through its less invasive and tailored techniques, patient outcome improvement, and elimination of complications while filling existing gaps in evidenced-based protocols.

## Materials and Methods

This systematic review employed a robust literature search to analyze the applicability of interventional radiology techniques in chronic pain control. The search was limited to articles published from 2020 to 2024, and the databases consulted were PubMed, Scopus, and the Cochrane Library. Key terms included interventional radiology, chronic pain, precision pain management, radiofrequency ablation, spinal injections, spinal cord stimulation, and neurolytic therapy. The searches were refined using Boolean operators such as AND and OR and manual scanning through the reference list of the identified articles.

Papers were selected using specific inclusion criteria. The inclusion criteria comprised RCTs, cohort studies, and case series published in English between January 2020 and December 2024 involving IR interventions for chronic pain patients. The primary objectives examined included pain reduction assessed by the Visual Analog Scale (VAS) scores, increased quality of life, and functional restoration. These were analyzed qualitatively regarding adverse events, physical and mental health, and patient satisfaction. The study excluded low-quality trials, cross-sectional reports, opinions or editorial pieces, languages other than English, and those focused on acute pain instead of chronic pain.

The articles were identified through a systematic search that followed the guidelines outlined in the PRISMA statement. An initial search produced 542 articles, and after eliminating the duplicates, 471 articles were included in the review process. Titles and abstracts were searched, and 102 articles were selected for full-text review. After careful review, 37 papers were selected and used in this review.

Ultimately, each reviewer completed a form independently to extract data from the studies involved in the meta-analysis. The reviewer extracted study information such as authorship, the year of publication, study type and number of participants, patient characteristics, IR techniques used, VAS scores, quality of life measures, functional rehabilitation, adverse effects, and satisfaction ratings. The research team discussed and resolved the extraction differences. Data synthesis involved both quantitative and qualitative analysis. The primary outcomes were the changes in mean VAS scores. In contrast, the data on the other secondary outcomes were described qualitatively to provide a comprehensive overview of the efficacy and safety of IR interventions.

The studies' quality was evaluated using validated instruments to increase inter-study comparability. The quality of the randomized controlled trials was assessed using the Cochrane Risk of Bias Tool, whereas the quality of the observational studies was assessed using the Newcastle-Ottawa Scale (NOS). Studies were then categorized into high, moderate, or low according to these evaluations. Finally, the quality of evidence was reviewed using the GRADE system, which evaluates limitations, including inconsistency, imprecision, and directness of the reports.

## Discussion

**Figure-1:**
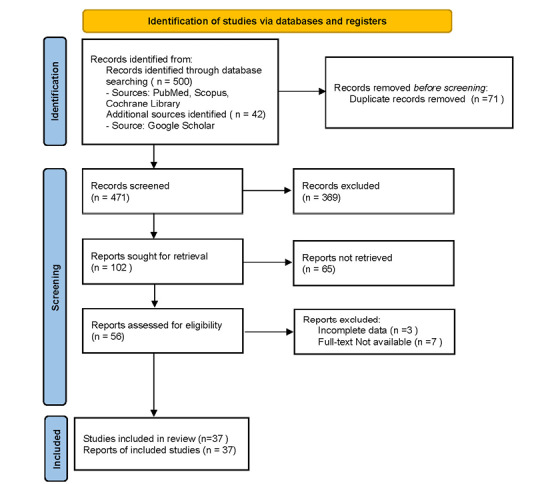
Figure 1. PRISMA Diagram

### 1. Pathophysiology of Pain and Role of Interventional Radiology

Pain is a broad concept referring to the physical and psychological experience that embraces the somatosensory, affective, physiological, and cognitive domains. This can be acute or chronic and has separate pathophysiological processes. Acute pain is a nociceptive response to noxious stimuli that signals possible or actual tissue injury [4]. It starts with nociception, where pain-detecting nerves called nociceptors identify potentially damaging mechanical, thermal, or chemical stimuli. These signals are conducted through A-delta fibers for sharp and localized pain and C fibers for dull and diffuse pain. Getting to the spinal cord, these signals are then conveyed through the somatosensory cortex for sensory awareness, the limbic system for subjective processing, and the prefrontal cortex for further analysis [4]. Acute pain usually is self-limiting and subsides when the affected tissue is healed, or the cause of the pain has been removed [35].

Chronic pain, on the other hand, is long-term pain that may last for months or years beyond the standard healing period of the body [5]. It is related to the concept of central sensitization, which is a condition where the nervous system becomes overly sensitive to signals. This enhances the dorsal horn of the spinal cord, NMDA receptors, and substances P and glutamate, which are excitatory neurotransmitters. Chronic pain presumably serves adaptive purposes, but its role is reversed, and it becomes pathological, hard-wiring pain signals that interfere with the patient’s well-being. Conditions like anxiety, depression, and social isolation are some of the mental health disorders that are often associated with chronic pain and add another layer to its management.

Interventional radiology (IR) encompasses a variety of procedures using image-guided approaches that reflect and complement an understanding of pain. Concerning peripheral pain mechanisms, invasive procedures like epidural steroid administration and nerve root blocks prevent nociceptive signal transmission and inflammation [6]. For example, epidural injections release corticosteroids surrounding inflamed spinal nerve roots that cure radiculopathy and reduce peripheral sensitization. In conditions that involve central mechanisms, techniques like spinal cord stimulation (SCS) & radiofrequency ablation (RFA) act as an upper level of neuromodulation [7]. SCS works by placing electrodes close to the spinal cord, generating electric currents to interfere with the pain messages before reaching the brain. RFA, on the other hand, utilizes heat to decommission targeted nerve fibers that transmit pain signals, such as in facet joint and sacroiliac joint pains [7].

Interventional radiology also plays an essential role in the treatment of visceral pain, especially in malignancy-associated disorders [8]. Innovative techniques such as celiac plexus neurolysis employ neurolytic procedures to block specific nerves that convey pain from the abdominal organs and significantly alleviate the suffering of patients with terminal cancer. These techniques are selective and exact, focusing on the areas of pain to give the best results.

Being able to focus on separate pain pathways, IR techniques allow for an individualized approach to pain therapy. Treatment plans can be personalised based on the onset and nature of the pain, the patient’s anatomical features, and other disorders. This increases the chances of successful treatment and reduces its side effects and risks, making IR part of personalized and targeted pain management. Finally, with the procedures being minimally invasive, many patients recover more quickly, and infection risks are minimized, making it an ideal option for patients who cannot undergo surgical procedures. In addition, utilization of IR techniques reduces the use of long-term opioid treatment, which is a significant issue in chronic pain management.

### 2.Techniques in Interventional Radiology for Pain Management

Interventional radiology (IR) has revolutionized pain management by offering minimally invasive, image-guided procedures that target specific pain mechanisms.

### 2.1. Spinal Injections (Epidural, Facet Joint, Nerve Root Blocks)

Lumbar epidural injections can be classified as standard or routine IR in the management of chronic pain, especially in conditions like radiculopathy, spinal stenosis, and facet joint arthritis. These injections release anti-inflammatory compounds like corticosteroids in the precise location of pain, thus decreasing inflammation and nociception pathways. Epidural Steroid Injections are employed for the management of radicular pain associated with nerve root entrapment. Research has established that epidural injections relieve pain in about 50-80 percent of patients, with the duration of the effects ranging from weeks to months [9]. The transforaminal approach can be beneficial, with pain relief being reported in 80% of patients with lumbar radiculopathy. Facet Joint Injections, on the other hand, are administered to directly address pain arising from the facet joints, which may result from arthritis or mechanical stress [10]. The overall success rate of these injections ranges from 60-75% and may be influenced by patient selection and injection techniques. Ultimately, a nerve root block is carried out for diagnostic and therapeutic reasons, especially when radiculopathy is suspected [11]. They offer short-term relief and point out the root of the problem causing pain.

### 2.2. Radiofrequency Ablation (RFA) for Facet and Sacroiliac Joint Pain

RFA is a low-risk procedure utilizing radiofrequency energy to destroy nerve cells that relay pain signals within the body. This is especially so in conditions affecting the facet and sacroiliac joints in the lower part of the spinal column [33]. In some cases, the relief has been reported to last up to one year after the treatment, and the success rate ranges between 60 and 80 percent. It is most effective for patients with well-defined pain syndromes and positive diagnostic blocks. Furthermore, RFA appears to offer considerable pain relief for SIJ dysfunction, with success rates that range from 70-75% [12]. It is commonly employed when other forms of treatment prove to be ineffective.

### 2.3. Spinal Cord Stimulation (SCS) for CRPS and FBSS

Spinal cord stimulation is a procedure in which electrodes are placed near the spinal cord to generate electrical stimulation that interferes with pain messages before they reach the brain. They work best for treating CRPS and FBSS and are also effective for managing localized forms of pain, such as neuropathic and cancer-related pain. Pain relief is achieved in 60-80% of patients with CRPS after SCS, which improves their quality of life. Addressing the problem at an early stage increases the likelihood of positive results. In patients with FBSS, SCS provides long-term pain relief and functional gains, while success rates are between 50% and 70% [13]. SCS not only eliminates the sensation of pain but also minimizes the use of opioids in treating patients with chronic pain, which is a significant concern in pain management.

### 2.4. Neurolytic Therapies for Visceral Pain

Percutaneous celiac plexus neurolysis is performed in severe chronic abdominal pain or pain from malignant diseases for pain management. These include using chemical methods to remove or block some networks of nerves that transmit pain. The Celiac Plexus Neurolysis technique has proved to provide good outcomes for pancreatic cancer pain with pain relief with a success rate of 70- 90% [14].

It also minimizes the need for opioids, enhancing patient’s quality of life dramatically. In managing pelvic pain, neurolysis is also offered on other related nerve plexus, particularly the hypogastric plexus. However, these procedures are considered only for patients with chronic pain and whose symptoms have not been relieved by conventional treatment.

Beyond the conventional methods of inquiry, innovation advancements in the field are broadening IR’s skill set. These include augmented reality (AR) and artificial intelligence (AI), which increase procedural accuracy and the ability to provide a prognosis, and robotic-assisted activities in surgery to avoid risks during complicated operations [24].

Moreover, features such as bariatric embolization that were initially used to control obesity are currently being piloted to address chronic pain [15]. In combination, these advancements illustrate the diversification and potential of IR in pain management, emphasizing its importance in acute and chronic pain management, reducing adverse events, and filling gaps in the pursuit of personalized pain treatment.

### 3.Comparative Effectiveness of Different IR Techniques

Interventional radiology depends on the disease being treated and the procedure performed for the therapy. Epidural steroid injections and nerve root blocks are helpful for radiculopathy, though they are used in different contexts. Nerve root blocks are used diagnostically and provide localized relief restricted to the area innervated by the specific nerve root [11]. In contrast, epidural injection is used in cases of general inflammation and provides more generalized pain relief. Like many procedures, the efficacy of radiofrequency ablation (RFA) versus spinal cord stimulation (SCS) varies based on the sort of pain. RFA is used for joint pain, especially facet joint or sacroiliac joint pain, which provides long-term relief with minimal invasiveness [12]. On the other hand, SCS can be more effective for conditions such as CRPS and FBSS or neuropathic pain because it targets neuronal pathways in the spinal cord. Neurolytic therapies, principally celiac plexus neurolysis, are particularly useful in the management of visceral pain arising from malignancy, where they are superior to systemic opioid analgesia [34]. However, due to the invasive nature of neurolytic procedures, they are only performed when other treatments have been unsuccessful. This selective and strategic use of IR techniques demonstrates the potential of these interventions for various types of pain and their ability to provide the best treatment options for patients depending on the specific nature of their case.

The effectiveness of IR in managing pain is variable. It depends on the type of pain syndrome the patient presents with and the measure of relief the treatment expects for any given period. Temporary treatment interventions such as epidural steroid injection and nerve blockage offer maximum results and can help manage pain within 3-6 months. These techniques are most effective in the emergency management of chronic pain conditions where there is a sudden worsening of pain and reduced functioning. Conversely, radiofrequency ablation (RFA) affords long-term pain relief, especially for facet and sacroiliac joint pain, with many patients reporting persistence of effects for 12 months or even more [16]. Equally, Spinal Cord Stimulation (SCS) demonstrates long-term efficacy for neuropathic pain disorders such as CRPS and FBSS. Most SCS users claim further improvement each year for 2 to 5 years after the implantation, demonstrating its effectiveness in modulating pain at the central level [17]. Neurolytic procedures like celiac plexus neurolysis can provide long-term analgesia for malignant visceral pain but vary with the stage of the disease.

Moreover, the long-term results of interventional radiology interventions are even better when combined with additional treatment options such as physiotherapy and psychotherapy, essential in treating chronic pain and helping the patient regain their quality of life and functionality. These findings emphasize the need to develop patient-tailored interventions for pain management to enhance their effectiveness for various groups of patients.

### 4. Safety Profiles and Complication Rates

IR procedures could be defined as minimally invasive procedures performed under image guidance, and the complication rates associated with them are relatively low compared to those of conventional surgical operations. Some of the known side effects include mild bleeding, infections, or brief discomfort at the site of the injections. Possible side effects identified for epidural steroid injections include postural puncture headache (0.6% for caudal epidural injections and 0.5-1% for lumbar interlaminar injections), and rarely, transient nerve involvement may occur [18]. Nevertheless, RFA has been associated with a low complication rate, with few skin burns or transient paresthesia reported [19].

Additionally, although SCS has been proven effective in providing significant pain relief, it is associated with potential complications, with lead migration reported to occur in 10-20% of patients and infection in 2-5% of patients [20]. Ultimately, while neurolytic therapies, like celiac plexus neurolysis, appear to be less painful, they are linked with a slightly higher risk of side effects, including transient diarrhea and hypotension, mainly when used for cancer-related pain. However, thanks to the noninvasive application of IR techniques, the rate of serious complications is much lower compared to traditional surgery approaches.

### 5. Individual Patient Factors in Interventional Radiology

Another aspect that is presumed to be a critical factor in the success of IR interventions is the characterization of the patients, such as the origin of pain, coexisting diseases, and anatomical factors. The type of pain, neuropathic, nociceptive, or visceral, determines the technique used. For instance, chronic neuropathic pain, such as Complex Regional Pain Syndrome (CRPS), is effectively treated with SCS, while mechanical/nociceptive pain caused by joint abnormalities is treated with RFA [22]. Sympathetically mediated visceral pain from malignancies sometimes requires neurolytic measures such as celiac plexus neurolysis. Additional conditions, including diabetes, cardiovascular disease, and coagulopathies, must also be considered when planning the treatment. This underlines the importance of post-therapy patient supervision since diabetics experience impacted pain tolerances and potentially diminished healing rates. It is essential to avoid the application of specific contrast agents in cardiovascular conditions, and coagulopathies may need strict anticoagulation control during procedures to reduce bleeding risk. Another factor is anatomical, which concerns changes in nervous and vascular supplies and the placement of internal organs. For instance, those with spinal deformities or prior surgeries may present with challenging access routes to epidural injection or nerve blocks, which necessitate the use of advanced imaging techniques [21]. These assessments have been essential to ensure that IR interventions are effective and safe from complications.

Personalized pain treatment is one of the central precepts of the IR approach. This strategy incorporates clinical, anatomical, and psychosocial aspects to achieve the best possible results [23]. For instance, patients with localized joint pain in whom diagnostic blocks have been positive are good candidates for RFA, while those with diffuse neuropathic pain may have more benefit from SCS. Customized treatment plans also consider specific patient preferences and ways of living. For example, patients with physically demanding work might opt for procedures with faster recovery periods, like nerve root blocks and epidural injections. However, patients with advanced cancer may be more willing to accept pain as a prominent symptom rather than undergo a more invasive procedure, which further supports neurolytic therapies. Advancements in Information technology, including artificial intelligence and machine learning, further amplify the possibility of personalizing IR interventions. These tools use big data to estimate the probability of a desired outcome and incorporate specific patient characteristics into treatment plans [24]. Furthermore, molecular-based diagnostics and biomarkers are under development for selecting the IR techniques that individual patients will benefit from the most.

### 6. Prospective Advantages of Precision Pain Management in Interventional Radiology

Effective pain management has become a crucial aspect of interventional radiology (IR), replacing conventional forms of pain management. As an image-guided procedure that spares healthy liver tissue, IR improves patient prognosis and fits within the concept of individualized medicine [25]. It also increases patient satisfaction and quality of life by easing early and appropriate chronic pain management through interventional radiology. In contrast to global approaches to chronic pain, IR methods are patient-specific and target specific pain pathways and pathophysiologic processes. For instance, spinal cord stimulation (SCS) can help increase the quality of life in 60-80% of patients with CRPS and FBSS [13]. Likewise, radiofrequency ablation for facet and sacroiliac joint pain provides durable pain relief in 60-80 percent of patients, allowing them to return to daily activities [16]. These outcomes not only cure the physiological pain sensations but also decrease the overall psychological costs of chronic pain, including anxiety and depression. Rehabilitating the patients and making them independent again are the goals of IR interventions, which help discharge patients more quickly and ensure a better quality of life and more satisfaction.

Personalized IR interventions are cheaper than routine surgeries and extended pharmacological treatments. The IR techniques are minimally invasive, translating to minimized hospital stays, less downtime for recovery, and lower healthcare costs [26]. For instance, epidural steroid injections and nerve root blocks, which offer temporary relief for a period of 3-6 months, are cost-effective and can eliminate some major surgeries. Likewise, RFA and neurolytic therapies provide long-term pain relief and limit the need for additional treatments and costs. A study compared SCS to the medical management of FBSS. It demonstrated a long-term cost benefit of SCS, where patients had fewer hospitalizations and improved quality of life over five years [27]. Moreover, the flexibility of applying interventions within the IR model sharply reduces the chances of complications that may increase healthcare costs. The cost-effectiveness implied by these features renders IR a feasible and widely appealing treatment choice regarding patient and healthcare system costs, especially with limited resources.

One of the most essential benefits of precision pain management within IR is offering less reliance on opioids as a treatment option [28]. Opioid use, primarily fueled by chronic pain, remains a critical factor in the international epidemic of opioid misuse. Many attractive IR options directly address the root of the issue, with encouraging efficacy and safety profiles, and may spare patients a lifetime of opiate dependency. For instance, neurolytic treatments, such as celiac plexus neurolysis, provide pain relief in 70-90% of malignancy-related visceral pain patients and also reduce opioid use [14]. Likewise, SCS efficacy in decreasing the use of opioids is demonstrated in patients with neuropathic pain, many of whom suffer from long-lasting pain relief without the need for medication [29]. This way, beyond the pain relief and reduction of patient suffering, IR interventions tackle the underlying causes of suffering and prevent the dangerous consequences associated with opioid use and misuse.

### 7. Knowledge Gaps and Future Directions in Precision Pain Management in Interventional Radiology

A significant gap in precision pain management within IR is the lack of a standard of care guideline encompassing evidence-based protocols [30]. Some of the current minimally invasive techniques include spinal injections, radiofrequency ablation (RFA), and spinal cord stimulation (SCS), which are effective at relieving chronic pain. Such variation can create disparity in patient treatment results and slow acceptance of effective practices across the health field. For example, in a systematic review of pain management programs, authors called for increased program standardization to guarantee patients equal access to adequate care [30]. These guidelines would create a straightforward method to identify which methods work for any given individual, offering high efficacy while ensuring safety. Creating these guidelines calls for extensive research and agreement from various specialists such as radiologists, pain specialists, and primary care physicians.

Unfortunately, few studies have been conducted to compare the effectiveness of different IR techniques. For instance, while RFA and SCS produce distinct outcomes in chronic pain, research comparing the efficacy of both methods for particular diseases such as CRPS or FBSS is needed. Such studies are critical in informing clinicians on the correct interventions to apply. Additionally, many investigations concerning IR strategies have provided evidence of their efficacy only for short-term outcomes with scarce and inconsistent long-term data. Further examination is required to determine the sustainability of pain relief, functional enhancements, and quality of life at longer intervals [31]. For example, although RFA treatment lasts 12 months, its effectiveness beyond the year is doubtful. Significant gaps in the current literature also require filling to determine patients ideal for undergoing IR interventions. To address this, identifying predictors of treatment response in chronic pain patients would increase the accuracy of management approaches that target these areas. Furthermore, evaluating the effectiveness and safety of new technologies like augmented reality for performing procedures and molecular imaging for pain management is imperative.

The application of AI in enhancing IR for precision pain management brings unprecedented possibilities. This is because AI can sort through vast amounts of data to discover trends in treatment and use this information for better decision-making. For instance, analytic models have been developed for optimizing procedural planning, such as an ideal needle path for spinal injections or the likelihood of neurolytic treatments [24]. Additionally, AI can add value to imaging by refining source identification and abbreviating the procedure time. "IR-GPT," a foundation model, attempts to integrate pre-, intra-, and post-procedural support into real-time for IR procedures, thereby enhancing the efficiency of activities within the field [32]. Nonetheless, AI is still a relatively new phenomenon in IR, and issues like data harmonization and standardization, identification of reliable algorithms, and potential ethical pitfalls have to be solved.

## Conclusion

Precision pain management within the interventional radiology (IR) field signifies the shift from traditional methodologies to aim for precise characteristics of chronic pain to provide a patient-oriented, evidence-based approach. Chronic pain, which afflicts millions of people, requires fresh approaches because of the complex and systemic nature of the phenomenon. PLP interventions, including spinal injections, RFA, SCS, and neurolytic therapies, have proved effective for both short-term and long-term relief, as well as quality of life enhancement and less reliance on opioids. Thanks to imaging and diagnostic technologies, IR provides the highest level of accuracy and proposes treatment approaches based on the pain source, the presence of the comorbid conditions, and the overall anatomy of the patient. This patient-focused approach leads to improved outcomes, higher satisfaction rates, and decreased healthcare costs, illustrating the importance of IR in modern pain management.

However, there are limitations to the field of IR; for example, there is no transparent, standardized, protocol-driven approach to treatment with IR, and there is a dearth of data on long-term outcomes. More rigorous clinical research is needed to fill these gaps, including comparative effectiveness studies and research on ideal patient selection criteria. Nevertheless, using AI and algorithm-based solutions may also improve the effectiveness of IR interventions, and interdisciplinary training and clinical guidelines may help standardize IR practices and outcomes among different centers.

Future directions for chronic pain management can be seen in integrating IR techniques into a more comprehensive, multidisciplinary approach. In this way, in addition to the physical benefit, IR interventions offer unique and comprehensive approaches to CVL and rehabilitative needs, encouraging patients to foster functional recovery and enhance their quality of life. As the field of MPS continues to develop, IR stays on the cutting edge of precision pain management, providing relief to patients struggling with challenging and chronic pain. By the nascent nature of the sector, constant innovation, and joint efforts of physicians and other healthcare workers, IR has the potential to transform chronic pain treatment into a paradigm of precision medicine.

## Conflict of Interest

None.
